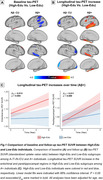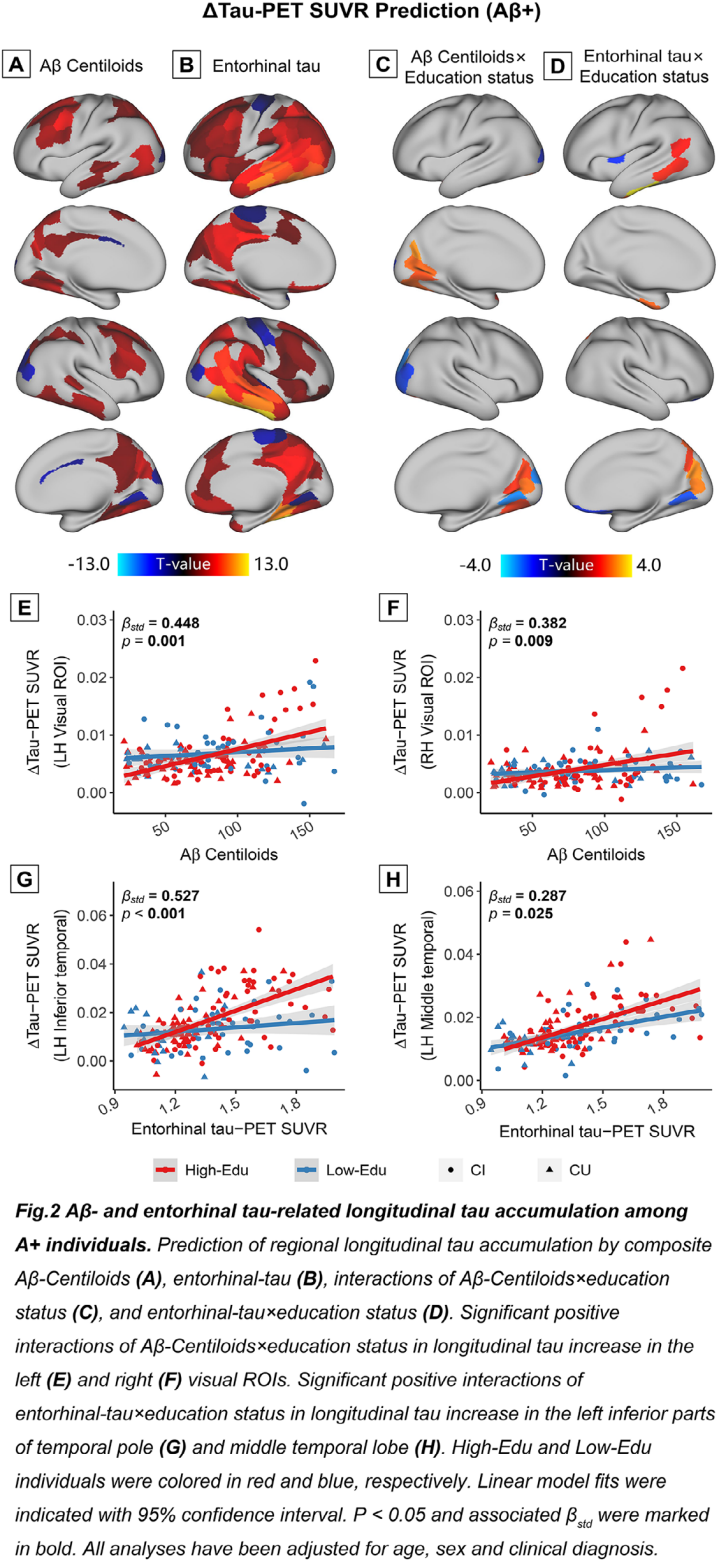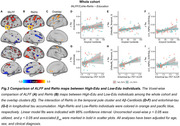# Higher educational attainment linked with accelerated longitudinal tau accumulation in Alzheimer’s disease

**DOI:** 10.1002/alz.091302

**Published:** 2025-01-09

**Authors:** Yue Cai, Xin Zhou, Lili Fang, Lin Liu, Anqi Li, Jie Yang, Pan Sun, Zhengbo He, Xianfeng Yu, Tengfei Guo, Alzheimer’s Disease Neuroimaging Initiative

**Affiliations:** ^1^ Institute of Biomedical Engineering, Shenzhen Bay Laboratory, Shenzhen, Guangdong China; ^2^ Tsinghua Shenzhen International Graduate School (SIGS), Tsinghua University, Shenzhen China; ^3^ Xuanwu Hospital of Capital Medical University, Beijing China

## Abstract

**Background:**

Previous cross‐sectional studies have extensively documented that higher educational attainment (EA) is associated with lower β‐amyloid (Aβ) plaques and tau tangles in Alzheimer’s disease (AD). However, studies investigating the relationship between EA and longitudinal tau accumulation are strikingly lacking.

**Method:**

We analyzed Aβ‐PET (A), tau‐PET (T), and 3D T1‐MRI images (N) from the ADNI cohort to identify 196 Aβ‐PET positive participants (A+) and 114 cognitively unimpaired participants without evidence of AD pathology and neurodegeneration (A‐/T‐/N‐/CU). A subset of 209 individuals had resting‐state functional MRI data (Rs‐fMRI), and 173 participants had at least one follow‐up tau‐PET. Participants were categorized into High‐Edu and Low‐Edu subgroups based on the median years of education of the whole cohort (16 years). Aβ‐PET SUVR of ^18^F‐florbetapir (FBP) or ^18^F‐forbetaben (FBB) were converted to Centiloids. Baseline and follow‐up tau uptakes based on the Schaefer‐200 atlas were compared between High‐Edu and Low‐Edu subgroups. The interaction of education status with baseline Aβ‐Centiloids and entorhinal tau on longitudinal tau accumulation was assessed. The voxel‐wise amplitude of low‐frequency fluctuation (ALFF) and Regional Homogeneity (ReHo) maps calculated from Rs‐fMRI data were compared between High‐Edu and Low‐Edu subgroups, and the impact of ALFF and ReHo alterations on Aβ‐Centiloids and entorhinal tau‐related longitudinal tau accumulation was also examined.

**Result:**

A+ High‐Edu individuals displayed lower baseline tau levels in temporal and parietal lobes but faster tau accumulation in medial and lateral temporal lobes (p<0.05, Figure 1). Moreover, A+ High‐Edu individuals revealed more pronounced positive associations between Aβ‐Centiloids and tau increases in bilateral visual cortices, as well as between entorhinal‐tau and tau increases in inferior parts of temporal pole and middle temporal lobe, compared to Low‐Edu individuals (p<0.05, Figure 2). Voxels with higher ALFF and ReHo in High‐Edu individuals converged in a cluster in the temporal pole, and the higher ReHo of this cluster predicted faster entorhinal tau‐related longitudinal tau accumulation in medial and lateral temporal cortices (p<0.05, Figure 3).

**Conclusion:**

These findings suggest that higher EA is associated with faster tau accumulation in medial and lateral temporal lobes in AD, and the accelerated tau accumulation of these regions may be linked to higher ReHo associated with education.